# Ultrathin Tunable Optomechanical Metalens

**DOI:** 10.1021/acs.nanolett.2c04105

**Published:** 2023-03-23

**Authors:** Adeel Afridi, Jan Gieseler, Nadine Meyer, Romain Quidant

**Affiliations:** † ICFO Institut de Ciencies Fotoniques, Mediterranean Technology Park, 08860 Castelldefels, Barcelona, Spain; ‡ Nanophotonic Systems Laboratory, Department of Mechanical and Process Engineering, ETH Zurich, 8092 Zurich, Switzerland

**Keywords:** Optomechanics, Reconfigurable metasurface, Nanophotonics, Metalens

## Abstract

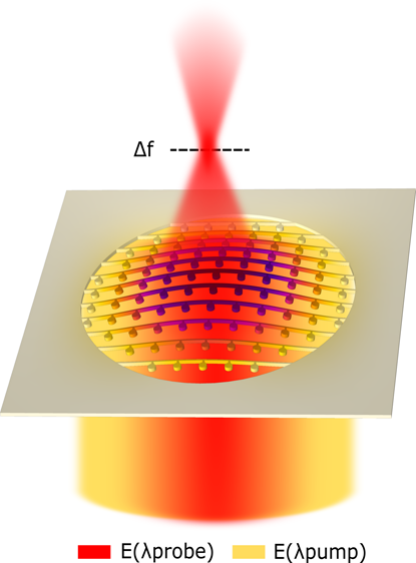

Reconfigurable metasurfaces offer great promises to enhance photonics technology by combining integration with improved functionalities. Recently, reconfigurability in otherwise static metasurfaces has been achieved by modifying the electric permittivity of the meta-atoms themselves or their immediate surrounding. Yet, it remains challenging to achieve significant and fast tunability without increasing bulkiness. Here, we demonstrate an ultrathin tunable metalens whose focal distance can be changed through optomechanical control with moderate continuous wave intensities. We achieve fast focal length changes of more than 5% with response time of the order of 10 μs.

A metalens is a two-dimensional (2D) metasurface^1−3^ that controls the amplitude, polarization, and phase of the impinging light using engineered sub-wavelength resonators, also known as meta-atoms (MA), judicially arranged in periodic or quasi-periodic arrays.^2,4−6^ To date, metalenses are the center of intense research efforts and have shown great potential to replace bulky optical elements, sometimes even with superior performance.^7^ However, the intrinsic passive nature of metalenses limits their use where active operation is required, such as adaptive vision and imaging.^5−12^ To address this challenge, various approaches have emerged that rely on changing the optical properties of the meta-atoms themselves or their surrounding medium.^6,12−26^ In particular, promising advances were reported using electro-optic control,^12,16−18^ temperature-induced effects,^19,20^ light-induced effects,^21−25^ phase change media,^26^ and mechanical actuation.^6,13−15^ Despite this progress, it remains challenging to combine compactness, fast operation speed, and low energy consumption. For instance, a phase-change material-based metasurface provides large tunability and ultrafast switch ON time (up to MHz) but suffers from long switch OFF time.^27^ Similarly, electrically controlled liquid crystal (LC) embedded metalenses operate at low driving voltages (<10 V). However, they require bulky LC chambers and are polarization-dependent due to the birefringence of the LC.^12,28^ Micro-electromechanical systems (MEMS)-based technologies promise large focal length changes and high switching speed but require high driving voltages.^6,15^ On the other hand, mechanical stretching requires bulky mechanical arms limiting integration and response time.^14^

Optomechanics,^29−35^ where optical forces mediate the interaction between light and structural mechanics, offers new possibilities for reconfigurable metasurfaces. When considering suspended meta-atoms patterned in a thin membrane, the enhanced electromagnetic forces at resonance can exceed elastic forces of the material, thereby inducing mechanical deformation. Already a small deformation alters the phase delay incurred by the impinging light substantially.^36^ First proposed theoretically,^37^ the concept of optomechanical metasurfaces features giant nonlinearity, optical bistability, and asymmetric light transmission. Shortly after, optomechanically induced modulation of light transmission through a metasurface was experimentally demonstrated.^38^ Similarly, gold meta-atoms supported by pairs of free-standing silicon nitride strings exhibit individual optomechanical plasmonic resonances.^39^ An experimental study of a suspended silicon carbide (SiC) metasurface supporting multimode vibrational resonances was reported.^40^ More recently, an experimental demonstration of nonreciprocal transmission and optical bistability at low intensity (∼10 W/cm^2^) in a free-standing Si_3_N_4_ membrane-based metasurface was presented.^41^ These observations suggest optomechanical control as a promising approach toward compact, fast, and low-power tunable metalenses.

In this Communication, we design, fabricate, and characterize an ultrathin optomechanically reconfigurable metalens operating in the near-IR regime. Our metalens is formed by an ensemble of suspended Huygens’ meta-atoms^42^ carved in a free-standing crystalline silicon membrane, as illustrated in Figure 1. The metalens is designed to focus the probe light at λ_probe_ = 1.31 μm while being controlled by a pump beam at λ_pump_ = 1.55 μm. Optical forces experienced by the meta-atoms translate into a mechanical deformation of the metalens (Δ*z*) and consequently a change in the focal length Δ*f*. Beyond demonstrating the tunability of the focal length by the pump light, we perform a full characterization of the lens performance including power dependence, focusing efficiency, and time response.

**Figure 1:**
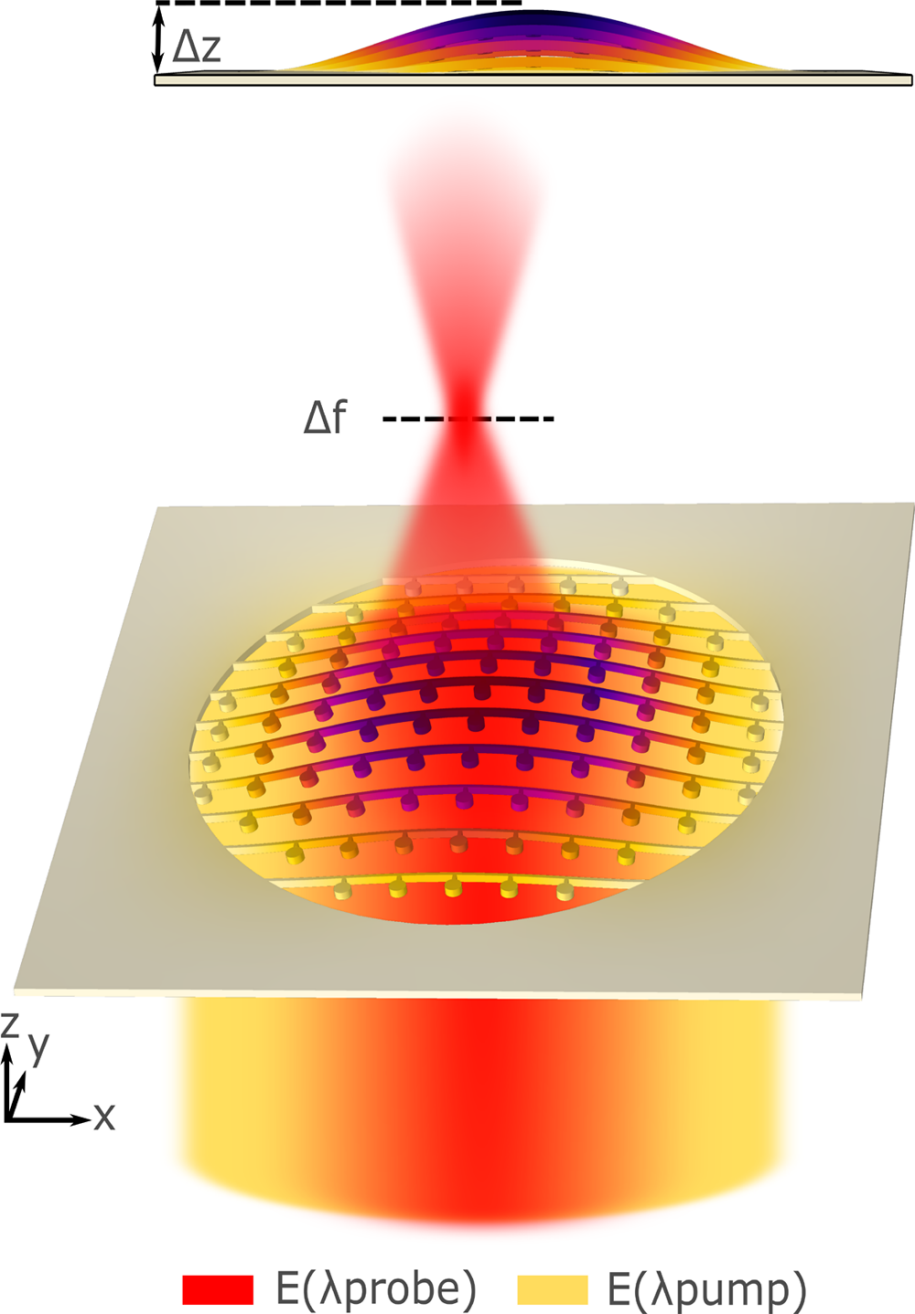
Artistic representation of an optomechanically reconfigurable metalens. The metalens is designed to focus light at λ_probe_. Upon illumination with a pump beam at λ_pump_, the resonantly enhanced optical force mechanically deforms the metalens by an amount Δ*z* resulting in a change of the focal length (Δ*f*) at λ_probe_.

Combining light focusing with optomechanical control requires careful multimode engineering at both pump and probe wavelengths. On the one hand, the set of meta-atoms must cover phase changes from 0 to 2π at λ_probe_. On the other hand, an additional mode at λ_pump_ is required to induce optical forces causing a sufficiently large mechanical deformation. To meet these requirements, we selected a rectangular periodic arrangement of suspended Huygens’ meta-atoms with periodicity *P*_*x*_ (*P*_*y*_) along the *x* (*y*) axis, in a 200 nm-thick silicon membrane. Each meta-atom consists of a disk with radius *r*, attached to a transversal nanobeam of width *w*_2_ via a short neck of width *w*_1_ (Figure 2a). Design optimization used a commercial finite element method simulator (COMSOL MULTIPHYSICS). Figure 2b displays the transmission *T* and reflection *R* for a specific meta-atom with *r* = 0.2 μm, *w*_1_ = *w*_2_ = 0.095 μm, and *P*_*x*_ = *P*_*y*_ = 1.275 μm. Under *x*-polarized light, the disk supports magnetic and electric dipolar resonances around λ_probe_ = 1.31 μm. The interaction between the electric and magnetic dipole resonances, controlled by the disk radius *r*, introduces a phase delay of the incoming light.^42,43^ Additionally, the disk attached to the supporting beam supports an electric mode around λ_pump_, which we use for optomechanical control.

**Figure 2:**
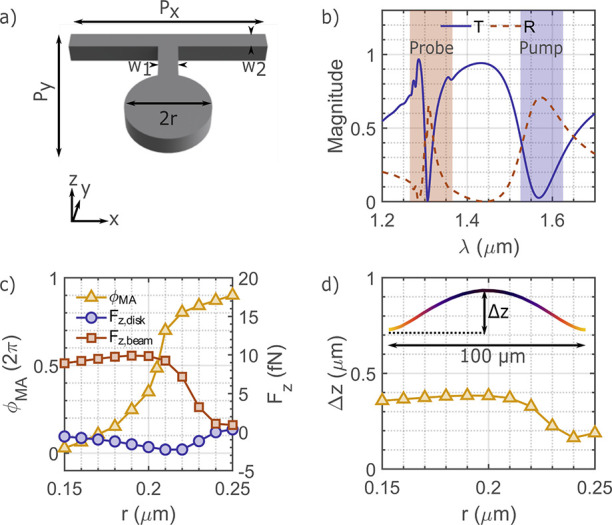
Geometry and simulation results of the silicon meta-atom. (a) Meta-atom geometrical parameters. (b) Simulated transmission *T* (blue solid line) and reflection *R* (red dashed line) of a meta-atom with disk radius *r* = 0.2 μm. The red (blue) shaded region signifies probe (pump) resonance. (c) Simulated phase due to the meta-atoms at probe wavelength λ_probe_ = 1.31 μm and calculated force experienced by the meta-atoms at pump wavelength λ_pump_ = 1.55 μm and pump intensity *I*_p_ = 1 μW/μm^2^, as a function of disk radius *r*. (d) Numerical simulation of the deformation Δ*z* for meta-atoms in a 1D array in the presence of pump light with *I*_p_ = 110 μW/μm^2^.

Next, we optimized an entire set of meta-atoms to provide 0–2π phase change at λ_probe_, by changing the disk radius *r* and beam width *w*_2_ simultaneously. The simulated phase imparted (ϕ_MA_) by these meta-atoms at λ_probe_ are shown in Figure 2c as a function of *r* (yellow solid line with triangular markers), while the respective *w*_2_ parameter is given in Table 1 in the Supporting Information. The periodicity and the width were kept constant at *P*_*x*_ = *P*_*y*_ = 1.275 μm and *w*_1_ = 0.095 μm, respectively.

In addition, we used the time-averaged Maxwell’s stress tensors to calculate the optical forces acting on the meta-atoms, as a function of the disc radius *r* (see Supporting Information for details). Figure 2c shows that the subunit (see Figure S4a in Supporting Information) of the meta-atoms experiences antiparallel forces along the *z*-direction, while the forces cancel out in the *xy* plane. The disk experiences a negative force that pulls against the illumination direction. Conversely, the beam is pushed by the incoming light. This force imbalance across the meta-atom introduces a tilt of the disk with respect to the beam, while the total positive force over a finite periodic array bends the whole array forward. Furthermore, we numerically simulated the deformation Δ*z*, for a maximum pump intensity of *I*_p_ = 110 μW/μm^2^ for each meta-atom arranged equidistantly in a one-dimensional (1D) array of 100 μm.^37^Figure 2d shows the deformation Δ*z* as a function of disk radius *r*. We observe a maximum deformation Δ*z* ≈ 380 nm for the meta-atom with *r* = 200 nm. Heating of the metalens under pump illumination induces thermal expansion and bending in the suspended meta-atoms. In order to quantify the thermal effects, we simulated the thermal distribution for each meta-atom using COMSOL MULTIPHYSIS (see Supporting Information for method). Figure S1b shows the temperature distribution for a meta-atom array with *r* = 0.22 μm for *I*_p_ = 110 μW/μm^2^. Similarly, Figure S1c shows the maximum temperature change Δ*T*_max_ for all the meta-atoms in the library. The maximum Δ*T*_max_ = 250 K occurs for the meta-atom with radius *r* = 0.21 μm. Based on the coefficient of thermal expansion of silicon,^44−46^ the thermally induced buckling of the array of length *L* = 100 μm is expected to be of only few nanometers. From these simulations, we conclude that the thermal expansion is negligible when compared to the optomechanically induced deformation.

To calculate the required phase profile ϕ_req_(*x*, *y*) in the *xy*-plane, which focuses light at focal length *f*, we used the hyperboloidal phase function.
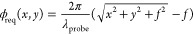
We fixed the focal distance and diameter of the metalens at *f* = 300 μm and *D* = 100 μm, respectively. We discretized the continuous ϕ_req_(*x*, *y*) with a suitable meta-atom such that, at a given *x* and *y* position, |ϕ_MA_ – ϕ_req_(*x*, *y*)| is minimized.

To fabricate our tunable metalens design, we employed top-down electron beam (E-beam) lithography. As material substrate we used a commercially available free-standing crystalline (100) silicon membrane from Norcada Inc. We spin-coated the membrane sample with the AR-P 6200.04 positive e-beam resist with a thickness of 230 nm followed by baking for 1 min at 150 °C. Afterward, E-beam exposure was carried out followed by one and a half minutes of development in an AR 600-546 developer at room temperature. We then etched the silicon membrane using HBr chemistry with an inductively coupled plasma (ICP) etcher. Finally, we stripped off the photoresist with an oxygen plasma etcher. Figure 3 shows scanning electron microscopy (SEM) images of the final metalens with a diameter *D* = 100 μm and designed focal length *f* = 300 μm.

**Figure 3:**
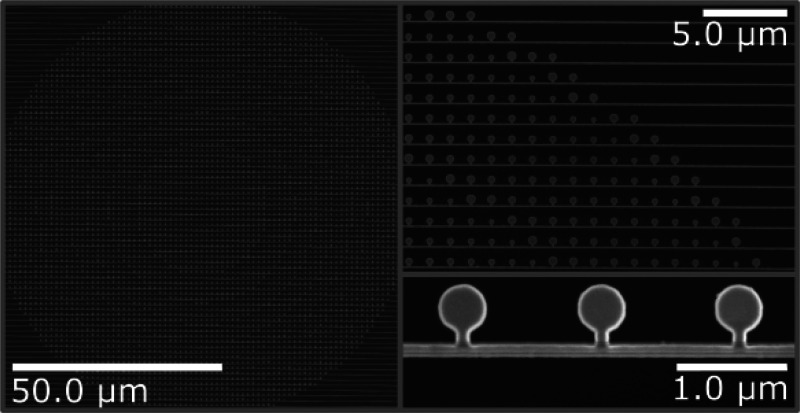
SEM micrographs of an optomechanically reconfigurable metalens. Fabricated metalens with diameter *D* = 100 μm and designed focal length *f* = 300 μm carved on a free-standing silicon membrane (Norcada Inc.) of thickness 200 nm.

We characterized the fabricated metalens using a homemade two-color optical setup (see Figure S2 of the Supporting Information). Collimated probe and pump beams are linearly polarized. The pump beam passes through a combination of an electro opto modulator (EOM), a polarizing beam splitter and a half-wave plate to control the power and polarization of the pump beam. Afterward, both beams were recombined on a 50:50 beam splitter. A 20× objective lens and numerical aperture (NA) = 0.4 focuses the probe and pump beam to a focal spot of 100 and 60 μm onto the metalens. A second identical objective mounted on a piezoelectric stage collects the light after the metalens. A band-pass filter with central frequency of 1.31 μm blocks the pump while allowing the probe to reach a near-infrared (NIR) camera and a photodiode. We acquired an image stack of 2D intensity maps along the principle optical axis with a step size of ∼1 μm by moving the collection objective with the piezo stage. First, we characterized the lens response in the absence of the pump beam. Figure 4a,b shows the 2D intensity map without pump (*I*_p_ = 0 μW/μm^2^) in the *xz*-plane (longitudinal) and at the focal plane (transversal), respectively. For the sake of space, we only present the axial intensity profile along the *xz*-plane. However, the focal length evolution is similar in the *yz*-plane, as the metalens confines the light in both *x*- and *y*-directions (see Figure 4b). The measured focal length is *f* = 328 ± 0.3 μm. We also measured the transmission efficiency of the metalens in terms of the focused probe intensity as a fraction of the incident probe beam intensity to ∼47%. Subsequently, we switched on the pump beam (intensity *I*_p_ = 110 μW/μm^2^) and observed a reduction of the focal length by Δ*f* = −20 μm. The 2D intensity maps (longitudinal and transversal) are displayed in Figure 4c,d. For further comparison, we plot the focus profile (*z*-cut) and 1D focal spot profile with and without pump laser in Figure 4e,f, respectively. It is noteworthy that the focusing quality and the full width at half-maximum (fwhm) value is well-preserved upon the introduction of the pump laser. Furthermore, the involved pump intensities are compatible with light-emitting diode (LED) arrays, thus eliminating the need for a bulky laser source.

**Figure 4:**
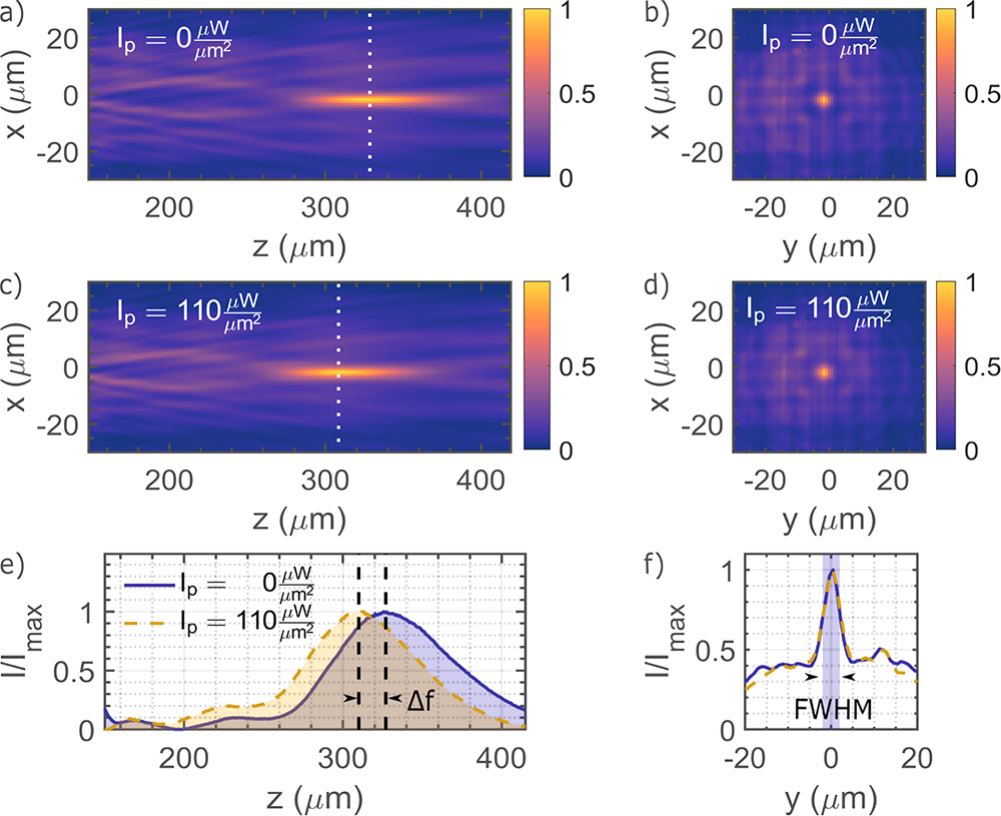
Optical characterization of the focal length with pump light ON and OFF. (a, c) 2D intensity maps in the *xz*-plane with the pump intensities *I*_p_ = 0 μW/μm^2^ and *I*_p_ = 110 μW/μm^2^, respectively. (b, d) 2D intensity profile of the focal spot in its respective focal plane (*xy*) under the pump intensities *I*_p_ = 0 μW/μm^2^ and *I*_p_ = 110 μW/μm^2^, respectively. (e) Axial optical intensity distribution for the two pump intensities. (f) 1D cut of the focal spot across the *y*-axis for the two pump intensities (blue solid line for *I*_p_ = 0 μW/μm^2^ and yellow dashed line for *I*_p_ = 110 μW/μm^2^. The fwhm is highlighted by the blue shaded area. All intensity profiles are normalized to the maximum probe intensity for the given pump intensity.

Although our metalens was optimized for *x*-polarized pump light, we also characterized the metalens under *y*-polarized pump light and found a focal change Δ*f* = −6 μm for the pump intensity *I*_p_ = 110 μW/μm^2^ (see Figure S3 in Supporting Information). This is consistent with numerical simulations shown in Figure S4b, which reveal that, under *y*-polarization, the effect of the pump on the beam becomes negligible, and, in contrast to the *x*-polarized case, the force on the disk is along the propagation direction.

When sweeping the pump intensity *I*_p_ from 0 to 110 μW/μm^2^, we observed a linear power dependence of the focal distance (Figure 5a). Another important parameter that characterizes the performance of a metalens is the focusing efficiency (FE), defined as the fraction of the transmitted power concentrated within an aperture of radius three times the fwhm of the focal spot at the respective focal plane of the metalens.^47^ We experimentally measured the FE for increasing *I*_p_ and observed a reduction from 78% (for *I*_p_ = 0 μW/μm^2^) to 68% (for *I*_p_ = 110 μW/μm^2^) as depicted in Figure 5a (blue circular dots).

**Figure 5:**
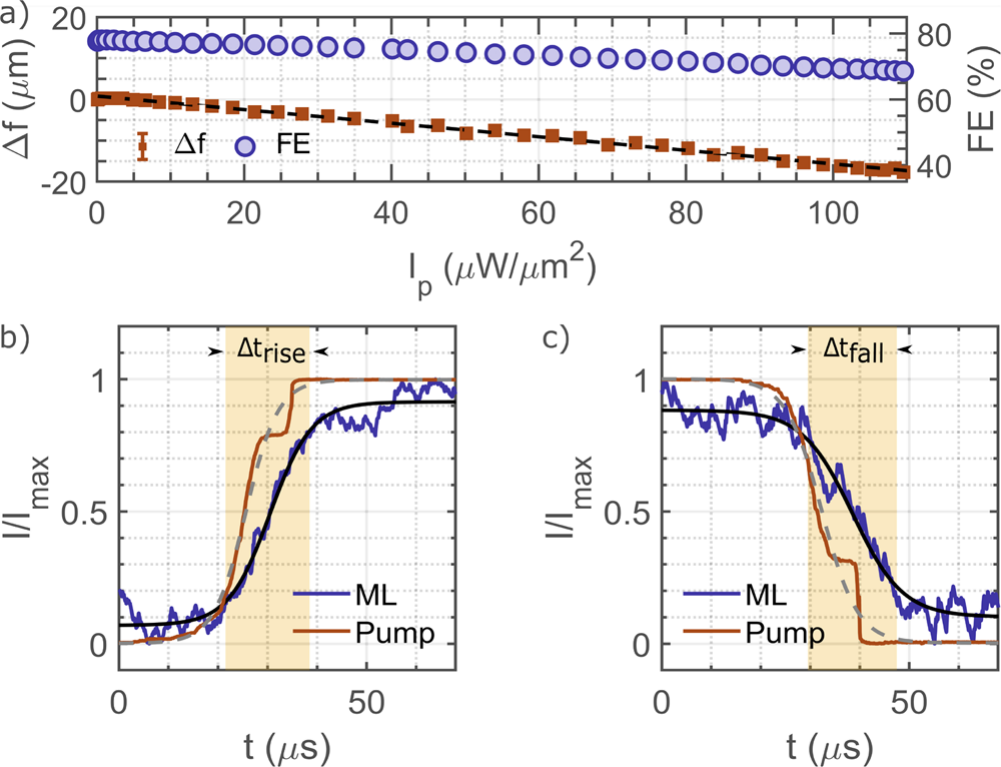
Focal length reconfigurability and switching dynamics of the metalens. (a) Focal length change and focusing efficiency (FE) as a function of pump intensity *I*_p_. Red dots represent experimental data while the black solid line is a linear fit. The blue circular dots show the focusing efficiency (FE). (b) Time response (rise time) of the metalens (blue solid line) switching from *f* = 328 to 308 μm under modulation of the pump beam from 0 to 110 μW/μm^2^ (red solid line). (c) Time response (fall time) of the metalens (blue solid line) switching from *f* = 308 to 328 μm under modulation of the pump beam from 110 to 0 μW/μm^2^ (red solid line). In (b, c), black solid line and gray dashed lines are sigmoid fits to the metalens and pump response, respectively. The 90% to 10% rise/fall time for the metalens is higlighted by the yellow shaded area.

Finally, we are interested in the switching dynamics of our optomechanically reconfigurable metalens. To measure its time response, we modulated the pump beam through the EOM with a square wave of frequency 0.5 Hz and amplitude 110 μW/μm^2^. The switching on rise time (10–90%) and switching off fall time (90–10%) of the EOM is limited to 14 μs. Figure 5b,c shows the time response of both the pump (red solid curve) and the metalens (blue solid line). We fit a sigmoid function to the data (black solid line for the metalens and gray dashed line for the pump) to obtain the rise and fall times. Our measurements give a rise time Δ*t*_rise_ = 17 μs (10% to 90%) and fall time Δ*t*_fall_ = 18 μs (90% to 10%) for the metalens highlighted by yellow shaded area in Figure 5b,c, respectively. From these values, we rule out that thermal effects dominate in the tunability of our lens. This conclusion is corroborated by the switching time calculated by Zhang et al.^37^

In conclusion, we proposed and realized an ultrathin varifocal metalens actuated by optomechanical control, which combines significant tunability with 10 μs response time. While the system was optimized to operate in the NIR region of the spectrum, extension to the visible frequency range could be achieved through an appropriate adjustment of the meta-atoms parameters and choice of their constitutive material. We foresee that future development of optomechanical integrated reconfigurable metalenses and other metasurface-based functionalities would greatly benefit from advanced mode engineering (exploiting for instance quasi bound states in the continuum), engineering the internal residual stress and exploiting the mechanical resonance frequencies of the system.

## Supporting Information Available

Additional figures, references, and discussion (ZIP)
Numerical simulations (optical and thermal) of meta-atoms, optical force calculation, meta-atom library, optical setup for characterization of metalens, metalens performance under *y*-polarization pump, optical forces and actuation under *y*-polarized pump, meta-atom actuation as increasing beam length and characterization of structural anisotropy (PDF)
