# Supplementary material for: Ultrathin Tunable Optomechanical Metalens
Source: Nano Lett. 2023 Mar 23;23(7):2496–501. doi: 10.1021/acs.nanolett.2c04105 (PMC10103287; doi:10.1021/acs.nanolett.2c04105)
Supplement: Supplementary file 2 — nl2c04105_si_002.pdf [file nl2c04105_si_002.pdf]

Supplementary Information For Ultra-thin Tunable Optomechanical Metalens

Adeel Afridi,[†,‡] Jan Gieseler,[†] Nadine Meyer,[‡] and Romain Quidant[*,‡]

†ICFO Institut de Ciencies Fotoniques, Mediterranean Technology Park, 08860 Castelldefels (Barcelona), Spain

‡Nanophotonic Systems Laboratory, Department of Mechanical and Process Engineering, ETH Zurich, 8092 Zurich, Switzerland

E-mail: rquidant@ethz.ch

Numerical simulation

Optical simulation

Optical response and mechanical actuation of the meta-atoms were numerically simulated using FEM based solver (COMSOL MULTIPHYSICS). For optical analysis and optimization, the RF module of the simulator was used, while the Structural Mechanics module was used for the mechanical analysis.

For optical simulation, a single unit cell (see Figure 2a in the main text) consisting of the suspended silicon meta-atom ($n = 3.2{+}0.04\mathrm{i}$[1]) in vacuum ($\epsilon_0 = 1$) was defined. Periodic boundary conditions in x and y directions were used and perfectly matching layer (PML) were used in $\pm z$ direction. The unit cell was illuminated using a plane wave (port boundary condition) with normal incidence. The unit cell was meshed with a step size of $\lambda/(20n)$, where λ is the wavelength of excitation and n is the refractive index. Finally the spectral

and phase response were extracted from the scattering parameters (S-parameters) of the port boundary condition.

Thermal simulation

We simulated temperature distribution for all meta-atoms in the library using the Heat Transfer module of COMSOL MULTIPHYSICS. We defined a $100\,\mu$m long 1D array of meta-atom (in x-direction) as shown in Figure S1a. The whole structure is enclosed by an air domain with temperature boundary conditions on each end and natural convention of air in the top and bottom of the domain. The absorbed power by the meta-atoms, for incident pump intensity $I_p = 110\,\mu\text{W}/\mu\text{m}^2$, is imported from the full-field optical simulations and used as heat source. Figure S1b shows temperature distribution for a meta-atom array with $r = 0.22\,\mu$m for $I_p = 110\,\mu\text{W}/\mu\text{m}^2$. Similarly, Figure S1c shows the maximum temperature change ΔT_{max} for all the meta-atoms in the library. The maximum $\Delta T_{max} = 250\,$K occurs for the meta-atom with radius $r = 0.21\,\mu$m. Based on the coefficient of thermal expansion of silicon,[2–4] the thermally induced buckling of the array of length $L = 100\,\mu$m is expected to be of only few nanometers. From these simulations, we conclude that the thermal expansion is negligible when compared to the optomechanically induced deformation.

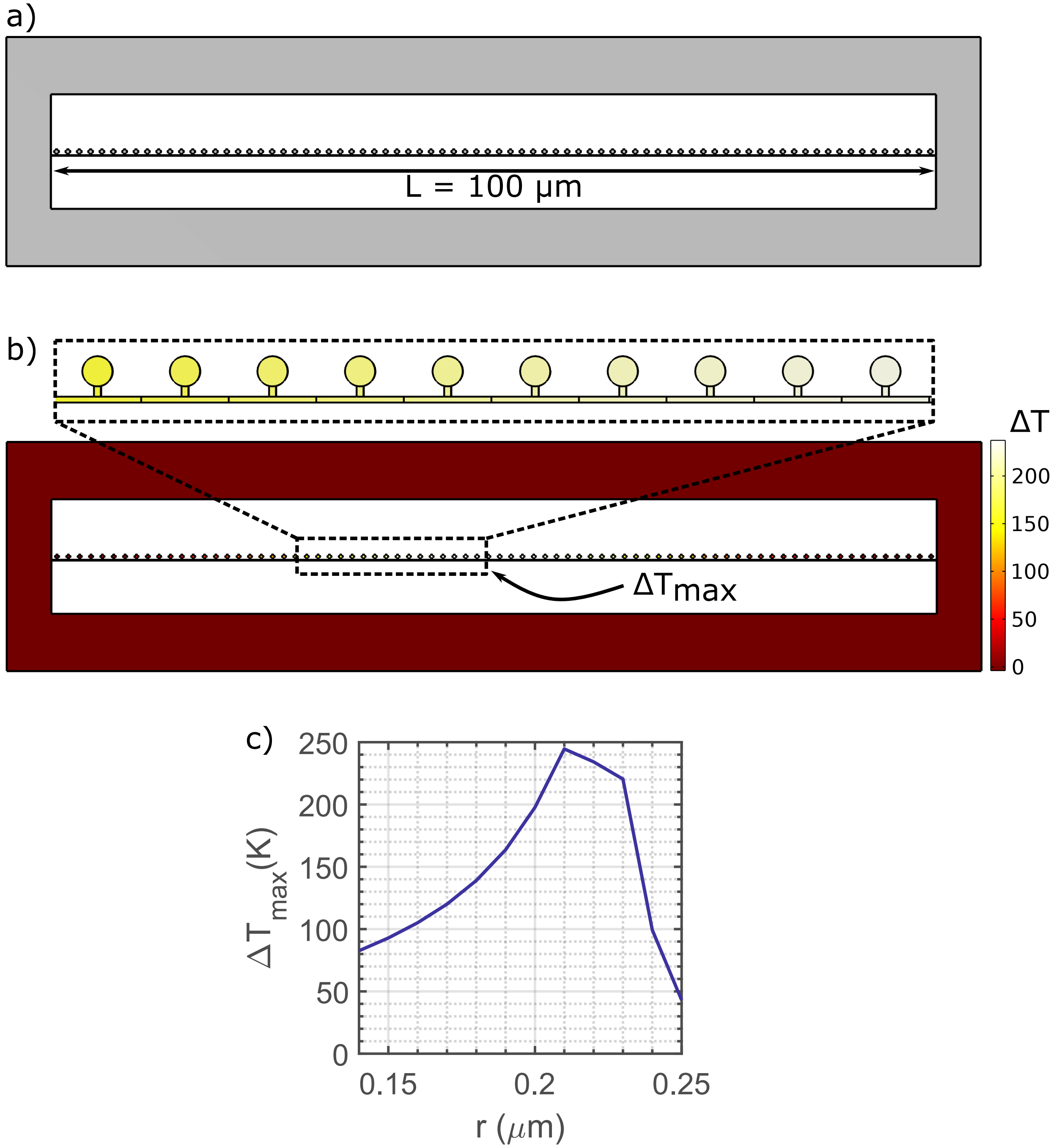

Figure S1: **Simulation of temperature distribution in the meta-atoms**: **a)** Schematic of the 100 μm long 1D array carved in an extended membrane. **b)** Simulated temperature distribution at $I_p = 110\,\mu\mathrm{W}/\mu\mathrm{m}^2$ in an array of meta-atom with $r = 0.22\,\mu$m (see Table 1 in supplementary information for other geometrical parameters). **c)** Maximum temperature difference ΔT_{max} as a function of the meta-atom radius r at pump intensity $I_p = 110\,\mu\mathrm{W}/\mu\mathrm{m}^2$. Where $\Delta T_{max} = T_{max} - 298$K.

Optical Force Calculation

In classical electrodynamics, the components of the time averaged, total force F, encompassing radiation pressure and gradient force, exerted by an optical field on a meta-atom can be calculated as a surface integral over the time averaged Maxwell's tensors:[5]

$$< F_i >= \oiint_S < T_{ij} > n_j dS$$

where S is the bounding surface around the meta-atom and n_j is the unit vector pointing out of the surface. We divided the bounding surfaces into two, i.e. around the disk and around the beam (see Figure S3a). $< T_{ij} >$ is the time averaged Maxwell's stress tensor:

$$< T_{ij} >= \frac{1}{2}\,\mathrm{Re}[\varepsilon\varepsilon_0(E_i E_j^* - \frac{1}{2}\delta_{ij}|E|^2)+$$

$$\mu\mu_0(H_i H_j^* - \frac{1}{2}\delta_{ij}|H|^2)]$$

where E and H are the electric and magnetic near field, respectively, extracted from the full wave optical simulation from COMSOL.

Meta-atom library

Table 1: Meta-atom library. Disk radius r and beam width w_2 are optimized together in order to acheive 0-2π phase change at λ_{probe} = 1.31 μm and enhanced optical forces at λ_{pump} = 1.55 μm.

Meta-atom library list												
r (μm)	0.150	0.160	0.170	0.180	0.190	0.200	0.205	0.210	0.220	0.230	0.240	0.250
w_2 (μm)	0.120	0.115	0.110	0.105	0.100	0.095	0.095	0.090	0.085	0.080	0.075	0.070

Optical setup

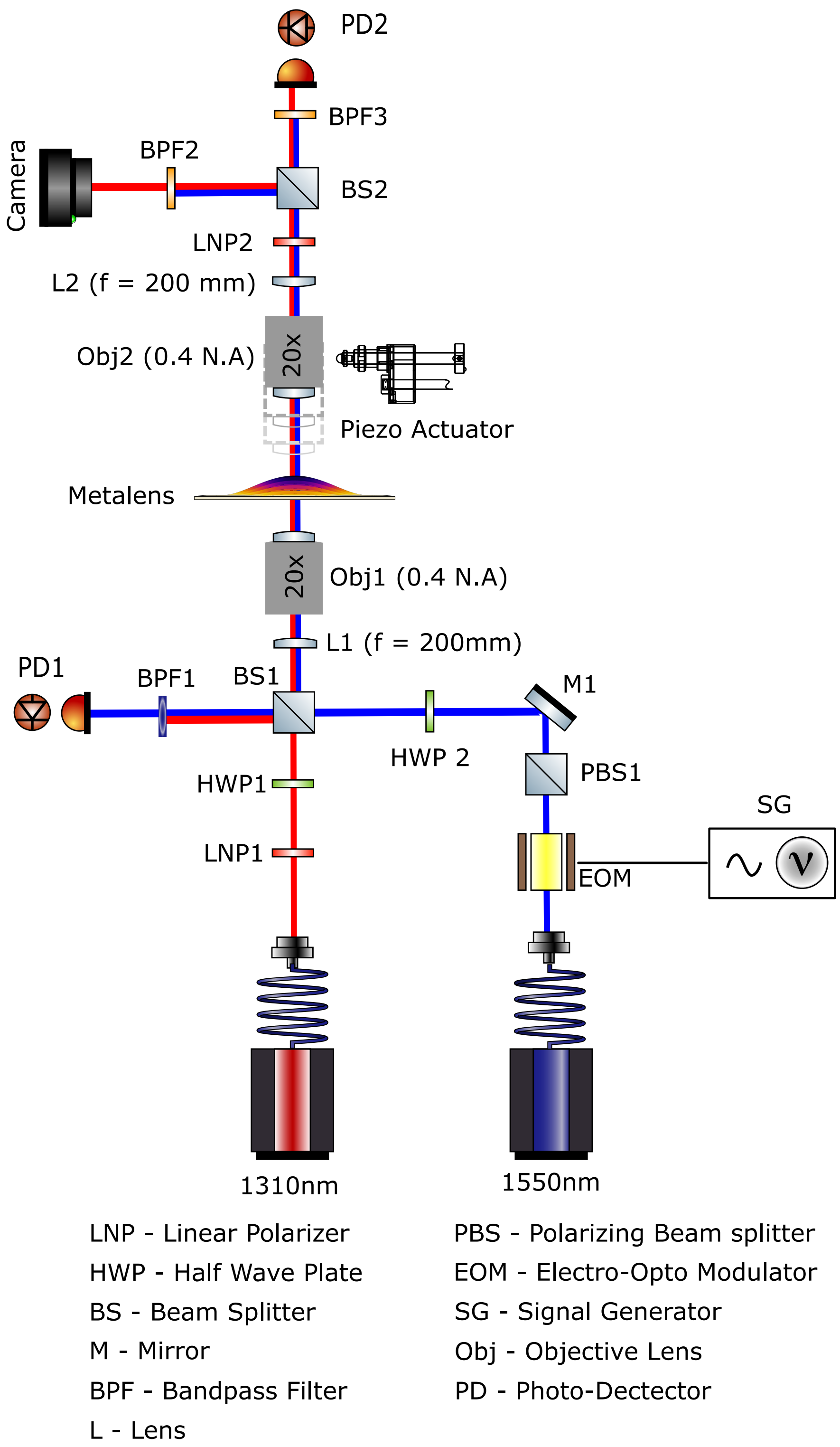

Figure S2: Schematic of the two colored optical setup.

Metalens performance under y-polarized pump

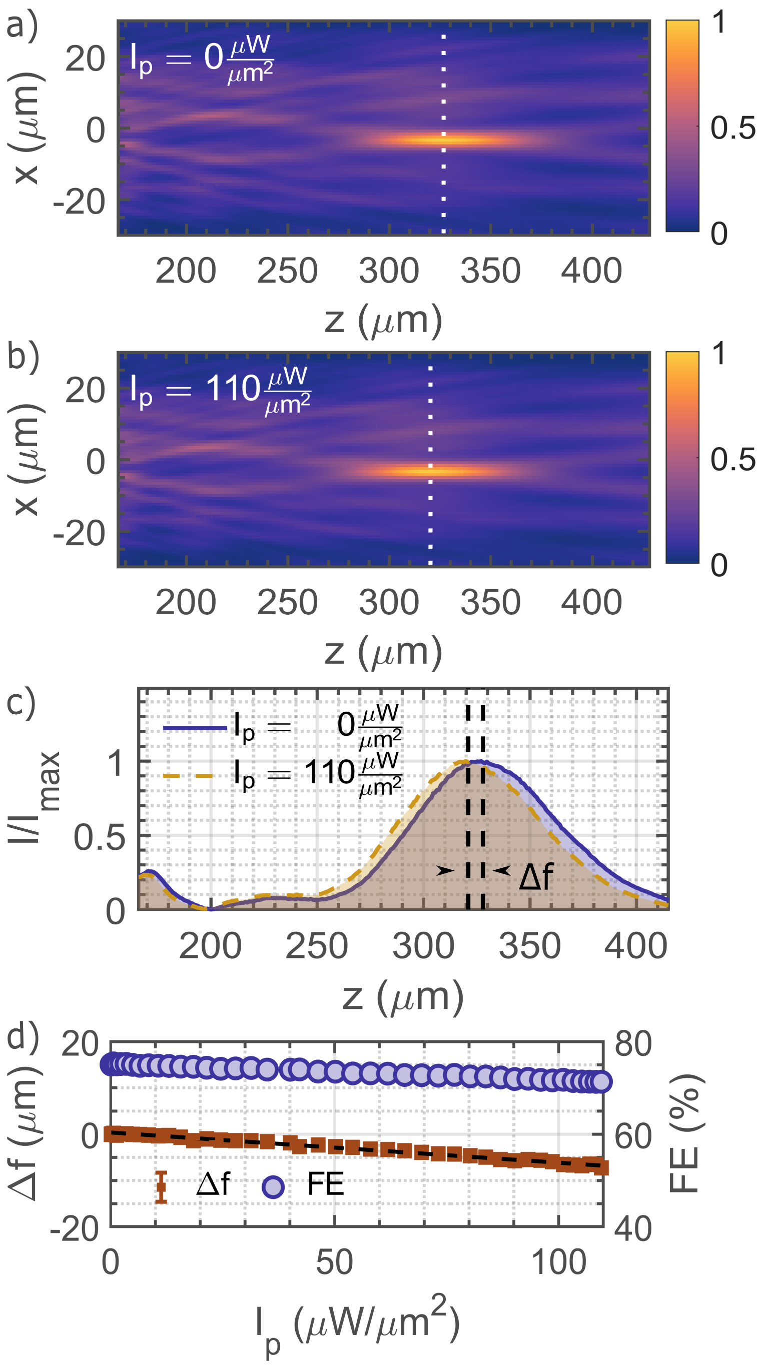

Figure S3: Optical characterization of the focal length reconfigurability with pump light y-polarized ON and OFF. **a)** and **b)** 2D intensity maps along xz propagation plane under the pump intensities $I_p = 0\,\mu W/\mu m^2$ and $I_p = 110\,\mu W/\mu m^2$, respectively. **c)** Axial optical intensity distribution for the two pump intensity states (propagation length z-crosscut). **d)** focal length change and focusing efficiency (FE) as a function of pump intensity I_p. Red dots represents experimental data while the black solid line is the linear fit and the blue circular dots show focusing efficiency (FE) in percentage.

Optical forces and actuation under y-polarized pump

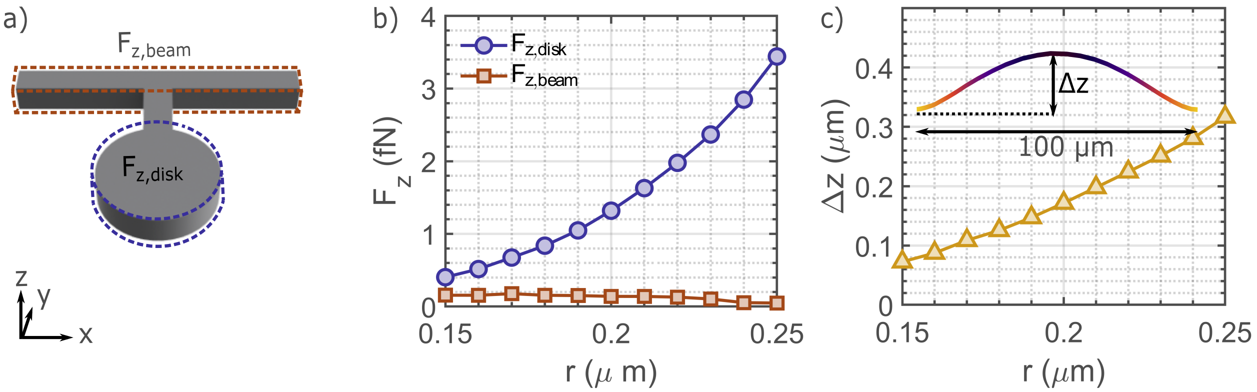

Figure S4: Forces and deformation simulation results of the silicon meta-atom for y-polarized pump light. **a)** Meta-atom unit cell indicating bounding surfaces for the force calculation. **b)** Calculated force experienced by the meta-atoms at pump wavelength $\lambda_{pump} = 1.55\,\mu$m and pump intensity $I_p = 1\,\mu\text{W}/\mu\text{m}^2$, as a function of disk radius r. **c)** Numerical simulation of the deformation Δz for a 1D meta-atom array of radius r in the presence of pump light with intensity $I_p = 110\,\mu\text{W}/\mu\text{m}^2$.

Meta-atom actuation as increasing beam length

In order to reduce the pump power requirement, we propose extending the supporting beam length by L_{ext} at both ends of the meta-atom array as shown in Figure S5a. For a meta-atom of radius $r = 0.2\,\mu$m and a pump intensity $I_p = 110\,\mu\text{W}/\mu\text{m}^2$, we simulated the mechanical deformation in COMSOL MULTIPHYSICS for increasing L_{ext}. The results depicted in Figure S5b show a linear increase of the maximum deformation Δz with increasing L_{ext}. For $L_{ext} = 20\,\mu$m, one can double Δz thus relaxing the power requirement by a factor of 2.

Characterization of structural anisotropy

We performed a controlled experiment to determine if any structural anisotropy exists in our metalens by flipping the device from front-to-back. Figure S6 compares the measured axial optical intensity distributions for the two orientations of the metalens and the two pump intensities $I_p = 0\,\mu\text{W}/\mu\text{m}^2$ (solid lines) and $I_p = 110\,\mu\text{W}/\mu\text{m}^2$ (dashed lines). It can

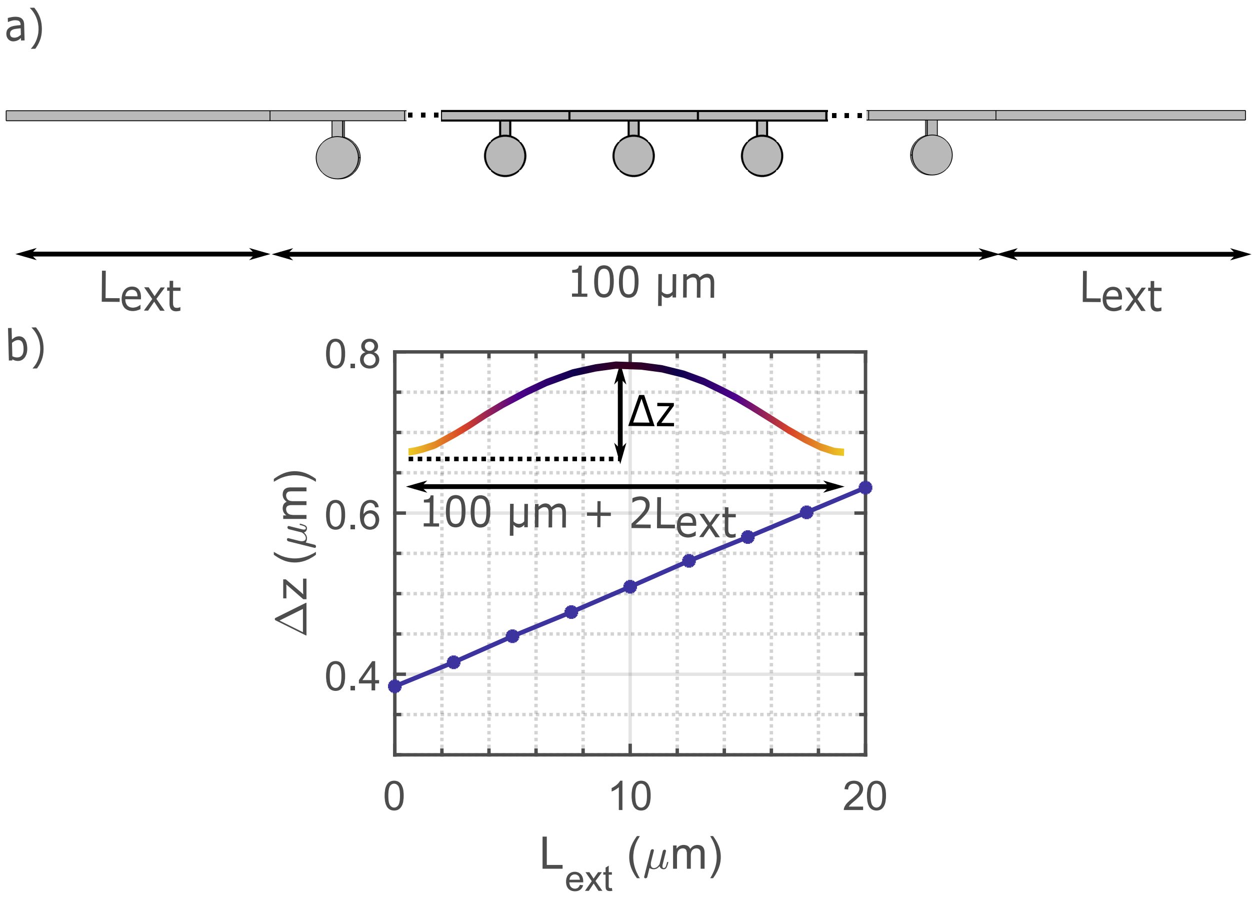

Figure S5: **Mechanical simulation of the meta-atom array with extended beams**: **a)** Schematic of the 100 μm long 1D array with extended beams at both ends. **b)** Simulated deformation Δz at I_p = 110 μW/μm^2 in an array of meta-atom with r = 0.2 μm as a function of L_{ext}.

be observed that the focal length change in both front (red lines) and back orientation (blue lines) is identical within the accuracy of our measurement. Based on this observation we deduce that the metalens is isotropic.

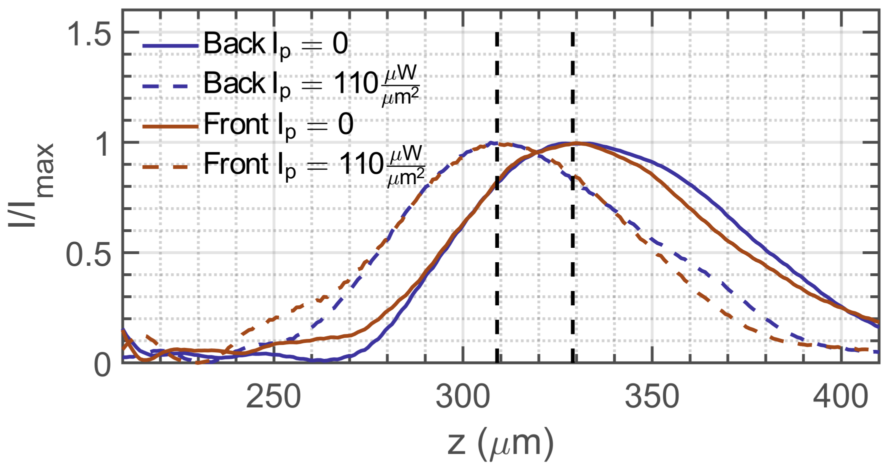

Figure S6: **Optical characterization of the metalens with pump ON and OFF and the device turned from front-to-back**: Axial optical intensity distribution for the two pump intensities $I_p = 0\,\mu W/\mu m^2$ (solid lines) and $I_p = 110\,\mu W/\mu m^2$ (dashed lines). The blue lines show the focal length evolution for the sample turned on its back with respect to the incoming light, while the red lines show the sample facing forward with respect to the incoming light.

References

(1) Karvounis, A.; Ou, J. Y.; Wu, W.; Macdonald, K. F.; Zheludev, N. I. Nano-optomechanical nonlinear dielectric metamaterials. *Applied Physics Letters* **2015**, *107*.

(2) Middelmann, T.; Walkov, A.; Bartl, G.; Schödel, R. Thermal expansion coefficient of single-crystal silicon from 7 K to 293 K. *Physical Review B* **2015**, *92*, 174113.

(3) Watanabe, H.; Yamada, N.; Okaji, M. Development of a laser interferometric dilatometer for measurements of thermal expansion of solids in the temperature range 300 to 1300 K. *International journal of thermophysics* **2002**, *23*, 543–554.

(4) Watanabe, H.; Yamada, N.; Okaji, M. Linear thermal expansion coefficient of silicon from 293 to 1000 K. *International journal of thermophysics* **2004**, *25*, 221–236.

(5) Jackson, J. Classical Electrodynamics, 3Wiley & Sons. *New York, NY, USA* **1998**,
