# Supplementary material for: Ultrathin Tunable Optomechanical Metalens
Source: Nano Lett. 2023 Mar 23;23(7):2496–501. doi: 10.1021/acs.nanolett.2c04105 (PMC10103287; doi:10.1021/acs.nanolett.2c04105)
Supplement: Supplementary file 1 — nl2c04105_si_001.zip [file nl2c04105_si_001.zip › Supplementary Information For Ultra-thin Tunable Optomechanical Metalens-Nanoletters-Final-Formatted.zip!/OpticalSetup.pdf]

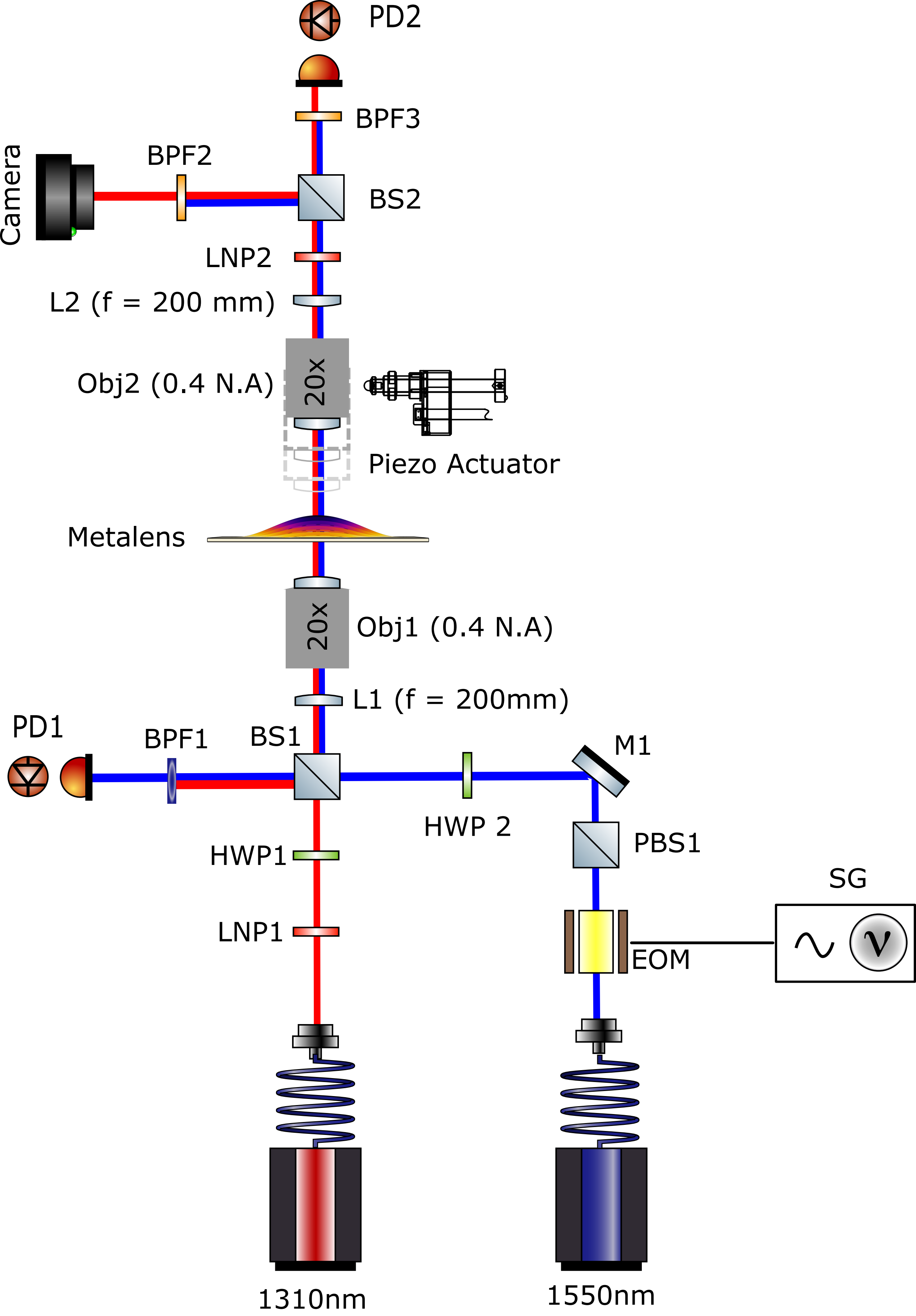

LNP - Linear Polarizer
HWP - Half Wave Plate
BS - Beam Splitter
M - Mirror
BPF - Bandpass Filter
L - Lens

PBS - Polarizing Beam splitter
EOM - Electro-Opto Modulator
SG - Signal Generator
Obj - Objective Lens
PD - Photo-Dectector
