# Supplementary material for: Ultrathin Tunable Optomechanical Metalens
Source: Nano Lett. 2023 Mar 23;23(7):2496–501. doi: 10.1021/acs.nanolett.2c04105 (PMC10103287; doi:10.1021/acs.nanolett.2c04105)
Supplement: Supplementary file 1 — nl2c04105_si_001.zip [file nl2c04105_si_001.zip › Supplementary Information For Ultra-thin Tunable Optomechanical Metalens-Nanoletters-Final-Formatted.zip!/thermo-mechanic.pdf]

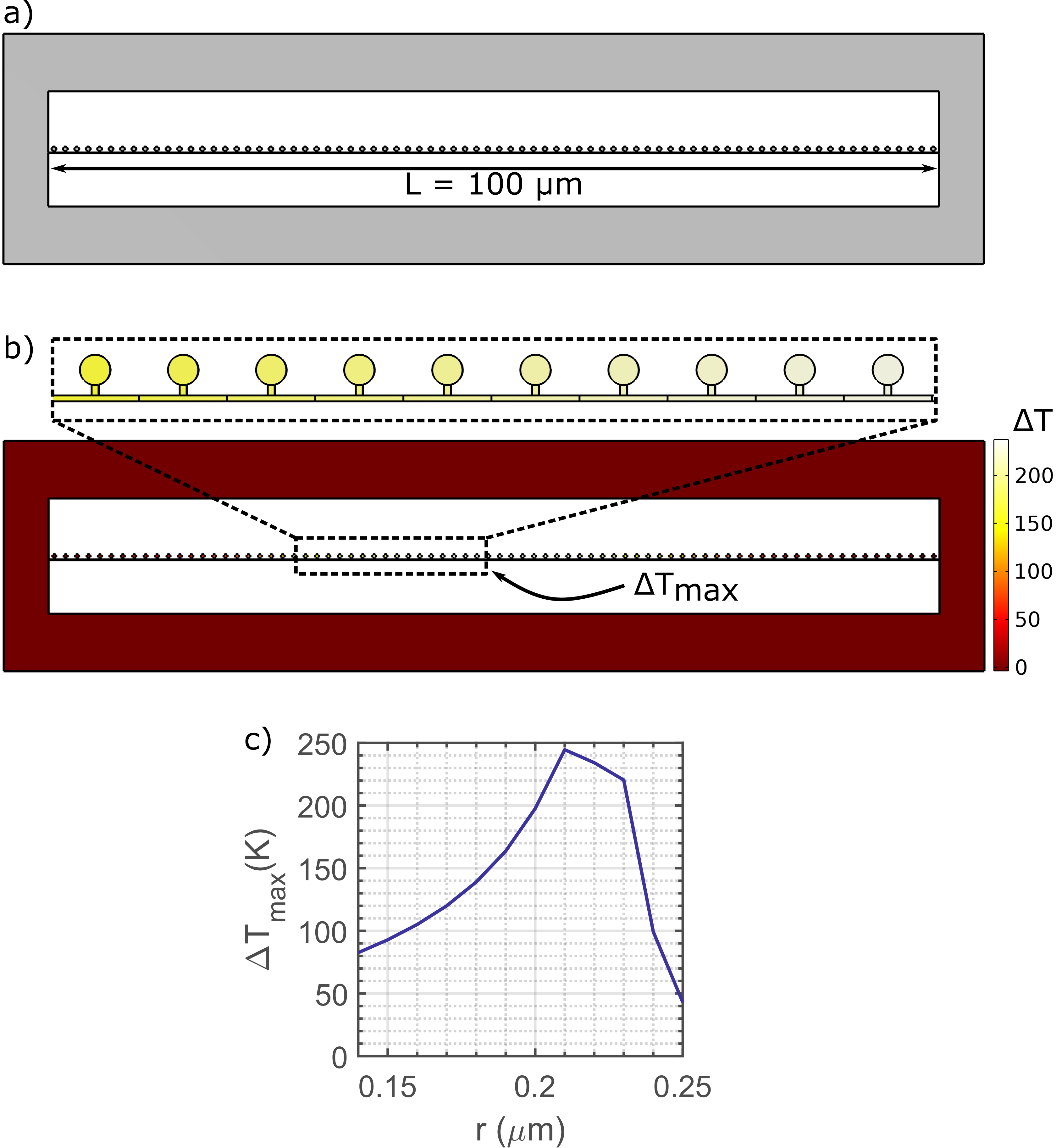
a)
L = 100 μm
b)
ΔT
200
150
100
50
0
ΔTmax
c)
250
200
150
100
50
0
0.15
0.2
0.25
ΔTmax(K)
r (μm)
