# Supplementary material for: Ultrathin Tunable Optomechanical Metalens
Source: Nano Lett. 2023 Mar 23;23(7):2496–501. doi: 10.1021/acs.nanolett.2c04105 (PMC10103287; doi:10.1021/acs.nanolett.2c04105)
Supplement: Supplementary file 1 — nl2c04105_si_001.zip [file nl2c04105_si_001.zip › Supplementary Information For Ultra-thin Tunable Optomechanical Metalens-Nanoletters-Final-Formatted.zip!/FigureR2.pdf]

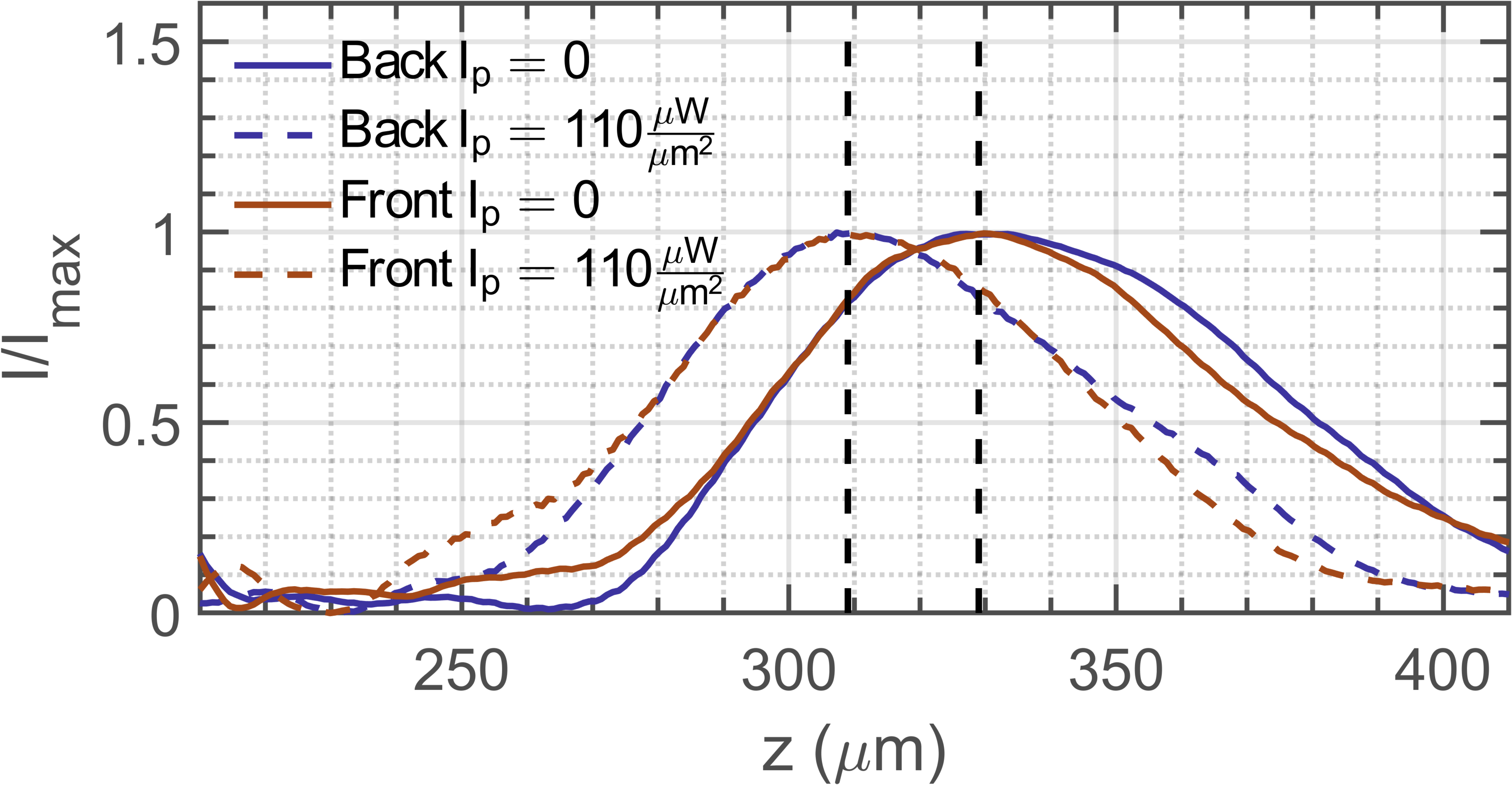

1.5
1
0.5
0
I/I_max
Back I_p = 0
Back I_p = 110 μW/μm²
Front I_p = 0
Front I_p = 110 μW/μm²
250
300
350
400
z (μm)
