# Supplementary material for: Ultrathin Tunable Optomechanical Metalens
Source: Nano Lett. 2023 Mar 23;23(7):2496–501. doi: 10.1021/acs.nanolett.2c04105 (PMC10103287; doi:10.1021/acs.nanolett.2c04105)
Supplement: Supplementary file 1 — nl2c04105_si_001.zip [file nl2c04105_si_001.zip › Supplementary Information For Ultra-thin Tunable Optomechanical Metalens-Nanoletters-Final-Formatted.zip!/FigureR3.pdf]

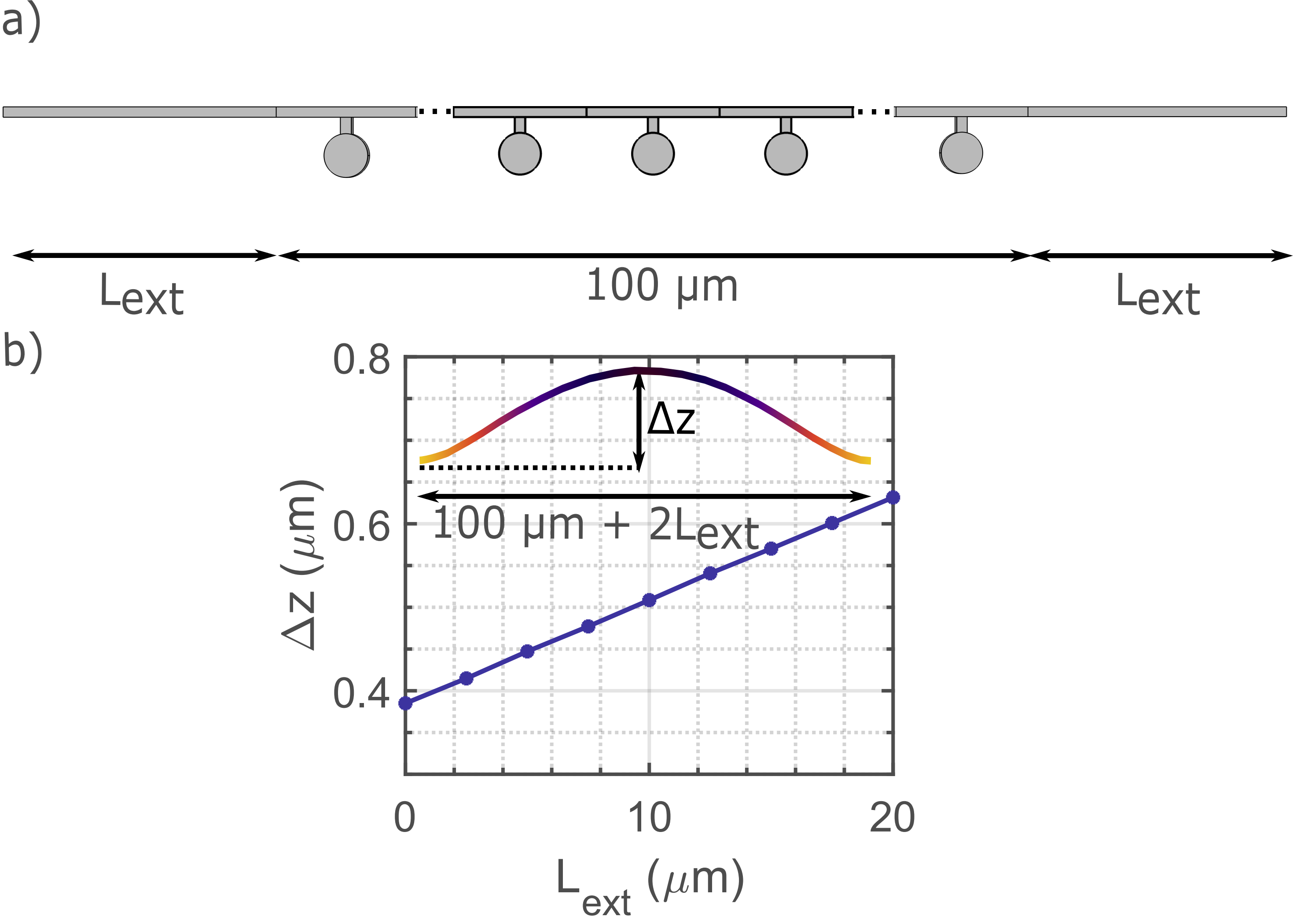

a)
Lext
100 µm
Lext
b)
0.8
0.6
0.4
Δz
100 µm + 2Lext
Δz (µm)
0
10
20
Lext (µm)
