# Supplementary material for: Ultrathin Tunable Optomechanical Metalens
Source: Nano Lett. 2023 Mar 23;23(7):2496–501. doi: 10.1021/acs.nanolett.2c04105 (PMC10103287; doi:10.1021/acs.nanolett.2c04105)
Supplement: Supplementary file 1 — nl2c04105_si_001.zip [file nl2c04105_si_001.zip › Supplementary Information For Ultra-thin Tunable Optomechanical Metalens-Nanoletters-Final-Formatted.zip!/FigureS2.pdf]

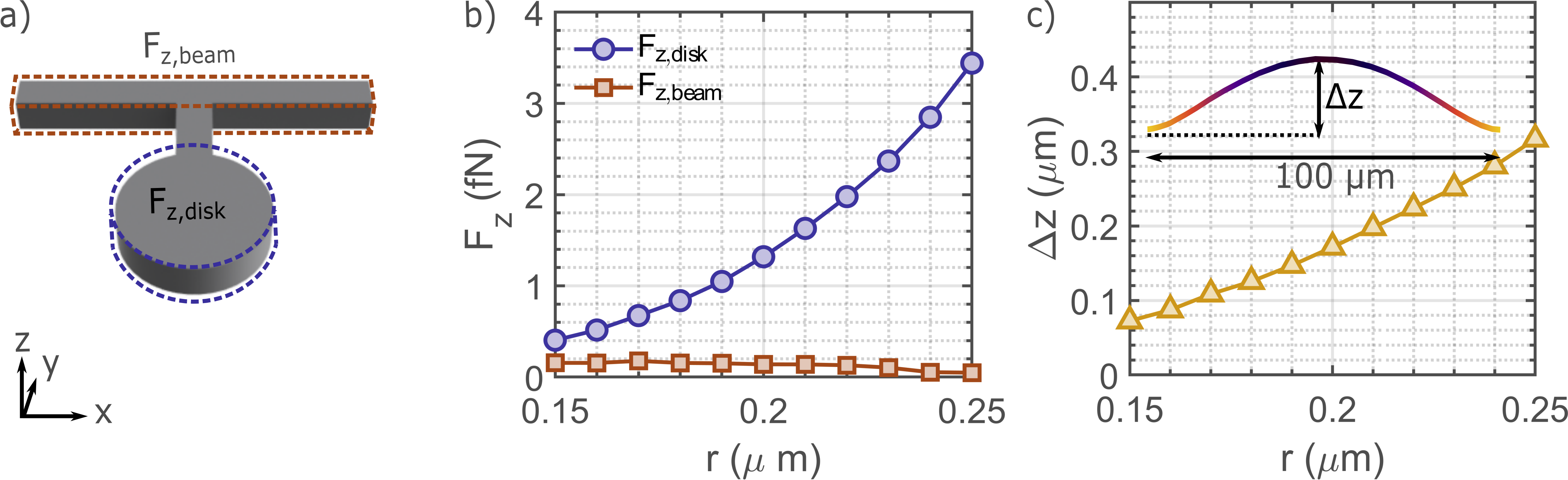

a)
$F_{z,beam}$
$F_{z,disk}$
z
y
x
b)
$F_{z,disk}$
$F_{z,beam}$
F_z (fN)
r (μ m)
0
1
2
3
4
0.15
0.2
0.25
c)
Δz
100 µm
Δz (μm)
r (μm)
0
0.1
0.2
0.3
0.4
0.15
0.2
0.25
