# Supplementary material for: Ultrathin Tunable Optomechanical Metalens
Source: Nano Lett. 2023 Mar 23;23(7):2496–501. doi: 10.1021/acs.nanolett.2c04105 (PMC10103287; doi:10.1021/acs.nanolett.2c04105)
Supplement: Supplementary file 1 — nl2c04105_si_001.zip [file nl2c04105_si_001.zip › Supplementary Information For Ultra-thin Tunable Optomechanical Metalens-Nanoletters-Final-Formatted.zip!/FigureS3.pdf]

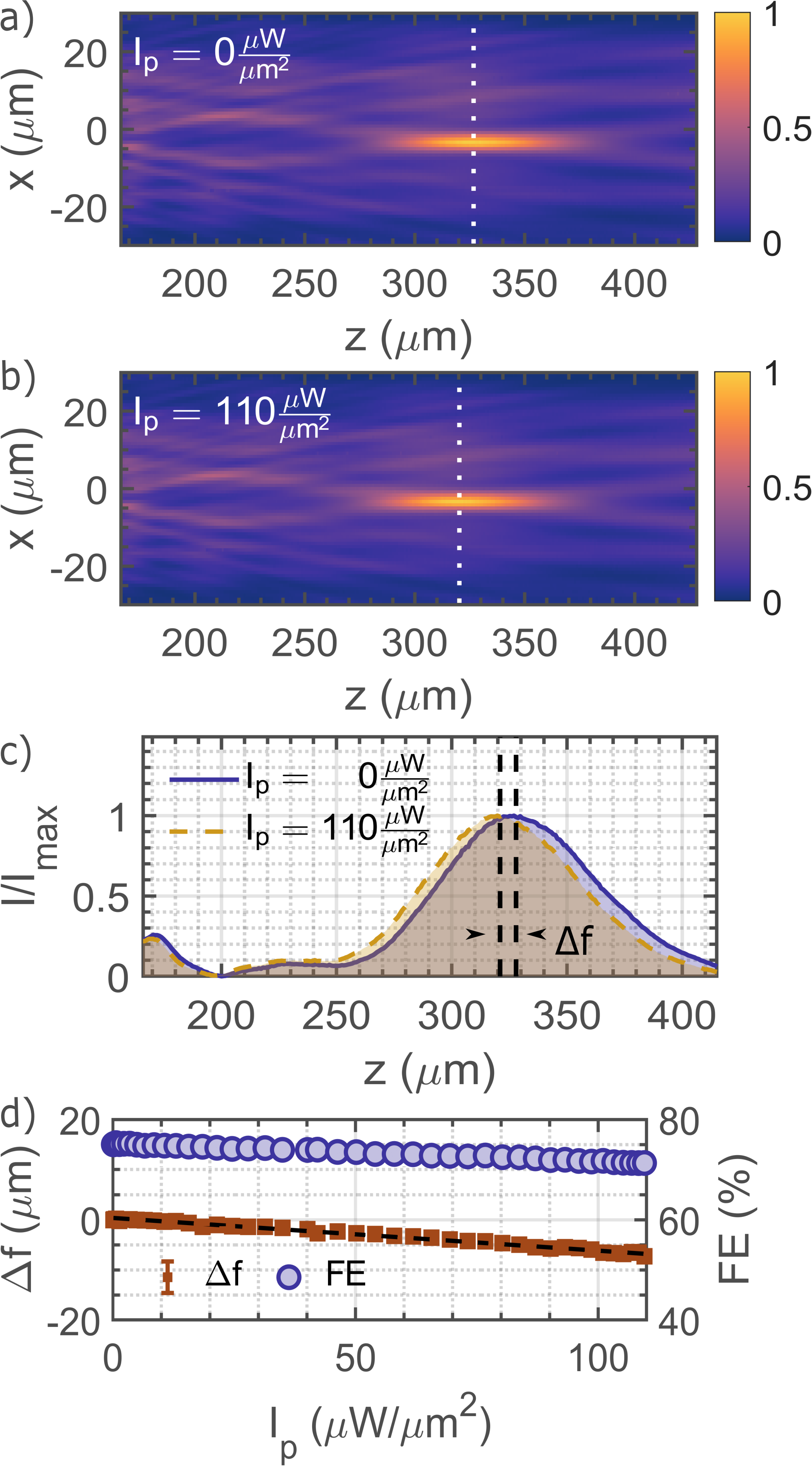

a)
$I_p = 0 \frac{\mu W}{\mu m^2}$
x (μm)
z (μm)
b)
$I_p = 110 \frac{\mu W}{\mu m^2}$
x (μm)
z (μm)
c)
$I_p = 0 \frac{\mu W}{\mu m^2}$
$I_p = 110 \frac{\mu W}{\mu m^2}$
Δf
I/I_{max}
z (μm)
d)
Δf
FE
Δf (μm)
FE (%)
I_p (μW/μm^2)
